# Cardiomyocyte Glucocorticoid Receptors Exacerbate Stress Effects in Myocardial Ischemia Injury in Mice

**DOI:** 10.3390/cells14242017

**Published:** 2025-12-18

**Authors:** Analilia Cardenas-Garza, Lilly A. Kamberov, Hemangini A. Dhaibar, Tanja Dudenbostel, Gopi Krishna Kolluru, Christopher G. Kevil, Robert H. Oakley, John A. Cidlowski, Luca Cucullo, Diana Cruz-Topete

**Affiliations:** 1 Department of Molecular and Cellular Physiology, Louisiana State University Health Sciences Center, Shreveport, LA 71103, USA; aca006@lsuhs.edu (A.C.-G.); ldk001@lsuhs.edu (L.A.K.); hdhaibar@gmail.com (H.A.D.); 2 Center for Cardiovascular Diseases and Sciences, Louisiana State University Health Sciences Center, Shreveport, LA 71103, USA; gopi.kolluru@lsuhs.edu (G.K.K.); chris.kevil@lsuhs.edu (C.G.K.); 3 Center of Excellence for Cardiovascular Diseases and Sciences, Louisiana State University Health Sciences Center, Shreveport, LA 71103, USA; 4 Department of Medicine, Huntsville Regional Medical Campus, University of Alabama at Birmingham, Huntsville, AL 35801, USA; tdudenbostel@uabmc.edu; 5 Department of Pathology and Translational Pathobiology, Louisiana State University Health Sciences Center, Shreveport, LA 71103, USA; 6 National Institute of Environmental Health Sciences—NIH, Research Triangle Park, Durham, NC 27709, USA; oakleyr2@niehs.nih.gov (R.H.O.); cidlows1@niehs.nih.gov (J.A.C.); 7 Department of Biological Sciences, Oakland University William Beaumont School of Medicine, Rochester, MI 48309, USA; 8 Department of Foundation Medical Studies, Oakland University William Beaumont School of Medicine, Rochester, MI 48309, USA

**Keywords:** stress, glucocorticoid receptors, sexual dimorphism, myocardial infarction, oxidative stress, cell death

## Abstract

**Highlights:**

**What are the main findings?**

- Systemic stress worsens ischemic injury in the heart through cardiomyocyte glucocorticoid receptor (GR) signaling.
- Cardiomyocyte GR acts as a sex-specific mediator of stress-induced cardiac damage.

**What are the implications of the main finding?**

- Identifies a potential mechanistic link between stress and adverse cardiac outcomes in women.
- Provides a foundation for developing targeted therapies addressing sex-specific cardiovascular risk.

**Abstract:**

An increase in mental stress is a recognized risk factor for cardiovascular disease (CVD). The present study investigated the relationships between stress, glucocorticoid receptors (GR), and ischemia/reperfusion (I/R) injury. We subjected male and female mice lacking cardiomyocyte GR (CardioGRKO) and their respective controls to a murine model of mental stress (restraint stress). Following stress exposure, mice from both experimental and control groups underwent I/R injury via surgical ligation of the left anterior descending coronary artery. Our findings suggest that the absence of cardiomyocyte GR mitigates the detrimental effects of restraint stress on infarct size and improves post-I/R survival rates in female mice. We found that cardiomyocyte GR deficiency protects the female heart from stress-induced damage by reducing oxidative stress (superoxide and lipid peroxide production). This study is the first to test the impact of systemic stress on cardiomyocyte GR activation, linking it to redox stress in the heart during I/R injury. Our findings provide proof of concept that stress exacerbates cardiomyocyte GR-mediated responses to myocardial infarction (MI) in the female heart. These insights may contribute to the development of sex-specific treatments and therapies tailored for women.

## 1. Introduction

Recent evidence suggests that myocardial infarction (MI) incidence is increasing among women aged 35–54 [1,2], with mental stress emerging as a prognostic factor associated with worse outcomes in premenopausal women [3,4]. The unexpectedly higher rates of MI and poorer post-MI recovery in young women compared to men [5,6] cannot be fully explained by traditional cardiovascular risk factors or sex hormone differences, suggesting that additional mechanisms such as stress-associated signaling in the heart may contribute to this disparity.

Psychological stress activates the hypothalamic–pituitary–adrenal (HPA) axis, which prompts the adrenal glands to release glucocorticoids. These hormones circulate throughout the body and exert their effects by binding to glucocorticoid receptor (GR), a ligand-activated transcription factor that regulates a wide arrange of gene networks [7]. Prior studies suggest that exposure to restraint stress exacerbates infarct sizes and increases oxidative stress in female mice [8]. Here, we tested the hypothesis that restraint stress worsens ischemia/reperfusion (I/R) outcomes in female hearts through GR signaling in cardiomyocytes. Using a transgenic mouse model lacking GR specifically in cardiomyocytes (cardioGRKO) [9], we investigated if deletion of GR reduces I/R injury in the context of stress in both female and male mice. This study builds upon prior work on stress–endocrine–cardiac interactions and provides proof-of-concept evidence that cardiomyocyte GR deletion mitigates stress-induced injury in the female heart. Our findings offer new mechanistic insight into GR-mediated, sex-specific susceptibility to I/R injury and point toward potential targets for future therapeutic strategies.

## 2. Materials and Methods

### 2.1. Reagents and Antibodies

Anti-Nrf2 (ab92946) and anti-4-hydroxynonenal (JAI-MHN-020P) antibodies were purchased from Abcam (Cambridge, UK) and Adipogen (Füllinsdorf, Switzerland). Dihydroethidium (DHE; cat. no. 37291) and dimethyl sulfoxide (DMSO; cat. no. 472301) were purchased from Sigma-Aldrich (St. Louis, MO, USA).

### 2.2. Animals

To generate the cardioGRKO mice the GR (Nr3c1) locus was modified by inserting loxP sites upstream of exon 3 and downstream of exon 4. Mice carrying the modified GR allele were then derived by blastocyst (albino B6) injection. Homozygous GR floxed (GR loxP, loxP; controls) mice were then mated with mice expressing Cre recombinase under the control of the alpha myosin heavy chain promoter (αMHCCre/+) to specifically delete GR in cardiomyocytes [9]. The resulting offspring were GRloxP/loxPαMHCCre/+ mice (designated cardioGRKO) and Cre-negative GRloxP/loxPαMHC+/+ (designated GR fl/fl) littermate mice that served as controls. Genotype was determined by PCR using DNA from tails as previously described [9]. All mice used in this study were on a C57BL/6J background. GR deletion efficiency and characterization were previously established by Oakley RH et al. [9] and our study used the same validated mouse line. One limitation of using cardioGRKO mice is their known cardiac phenotype, characterized by the development of pathological hypertrophy between 12 and 16 weeks of age. However, these mice do not display significant abnormalities before 12 weeks in males and 16 weeks in females [10]. To minimize the influence of this underlying phenotype, we performed baseline cardiac function testing at 7 weeks of age (prior to stress or IR exposure). Restraint stress/IR was initiated at 8 weeks, and after the 2-day reperfusion period the mice were ~10–12 weeks old, a time point that precedes the expected onset of overt hypertrophy. In addition, any cardioGRKO mice exhibiting cardiac abnormalities at baseline were excluded from the stress/IR protocol. Mice were categorized by sex (male and female) and genotype: control (GR fl/fl) or cardioGRKO. Female mice were housed in groups of four to synchronize their estrous cycle, and vaginal smear cytology was used to determine the phases of the estrous cycle. Surgical procedures were performed during the proestrus phase (high estradiol levels). Each genotype was further randomized by treatment into non-stressed and stressed groups, and each group was subsequently randomized into sham-operated (no ligation) and ischemia/reperfusion (IR; ligation for 30 min) subgroups. We initially allocated 60 mice to each NS and S group, which were further divided into 30 mice each for the sham and IR subgroups. However, not all animals survived the surgical procedure; some also died during the 48 h reperfusion period, either due to intubation or because they did not wake up after anesthesia. These animals were excluded from downstream experiments. The final sample sizes for the survival curves were non-stressed-control males = 20, non-stressed-control females = 22, stressed-control males = 20, stressed-control females = 18, non-stressed-CGRKO males = 19, non-stressed-CGRKO females = 20, stressed-CGRKO males = 20, and stressed-CGRKO females = 21. Sample sizes for downstream experiments varied from 3 to 10 animals per group as specified in each figure legend. 

During the stress challenge, control mice (non-stressed group) remained in their cages, which were covered and placed in a quiet laboratory area. Mice in the stress group were removed from their cages and subjected to repeated cycles of restraint stress for 5 consecutive days (six cycles per day; 60 min per cycle with 30 min intervals between cycles). The restraint procedure involved placing each mouse on a plastic platform and restricting their upper body and limb movement (excluding the head) using double-sided Velcro tape (Scotch fasteners; Velcro Companies, Manchester, NH, USA). The tail was also restrained using non-adhesive medical tape (Nexcare, St. Paul, MN, USA). During the stress cycles, non-stressed mice were also deprived of food and water to control for these variables. During the 30 min intervals between cycles, stressed mice were returned to their cages, and all animals (stressed and non-stressed) were given free access to food and water. At the end of each daily stress session, all mice were returned to the animal facility for overnight housing. The behavioral effects of this stress model have been previously validated by our group and are described in detail in our published work [8].

All experiments were approved and performed according to the Animal Care and Use Committee guidelines at LSU Health Sciences Center Shreveport (Protocol # P23-004, approval date 27 July 2022).

### 2.3. Left Anterior Descending Artery Ligation (LAD) Procedure

The ischemia/reperfusion (I/R) procedure was performed as we previously described [8]. Female mice were subjected to I/R at proestrus (high estradiol levels). Anesthesia was induced with 5% isoflurane (0.4 L/min O_2_) and maintained with 2% isoflurane. Following this procedure, the mouse was placed on a surgical platform, and the chest was shaved and prepared with betadine and alcohol before the chest incisions were made. The left anterior descending artery (LAD) was located and ligated. The occlusion of the LAD was confirmed by the appearance of a paler color on the anterior wall of the left ventricle (LV). After 30 min of ischemia, the occlusion was removed, reperfusion was confirmed, and the chest cavity was then closed. Sham-operated animals underwent the same surgical procedures, including thoracotomy and exposure of the LAD; a suture was passed under the artery but not tied to induce occlusion. All animals were continuously monitored for survival and signs of pain/distress for 48 h following I/R. Animals that did not survive the procedure or died before the 48 h reperfusion endpoint were excluded from downstream analyses and were only included in the survival curve.

The survivors were sacrificed, and blood and tissues were harvested for downstream analysis. All experiments were approved and performed according to the Animal Care and Use Committee guidelines at LSU Health Sciences Center Shreveport (Protocol # P23-004, approval date 27 July 2022).

### 2.4. Survival Analysis

Both non-stressed and stressed male and female mice—with either intact GR (GR fl/fl) or lacking cardiomyocyte GR (cardioGRKO)—were monitored daily for 48 h following ischemia/reperfusion (I/R) to record morbidity and mortality. Each group comprised the following numbers: non-stressed-control males = 20, non-stressed-control females = 22, stressed-control males = 20, stressed-control females = 18, non-stressed-CGRKO males = 19, non-stressed-CGRKO females = 20, stressed-CGRKO males = 20, stressed-CGRKO females = 21. Survival was analyzed using Kaplan–Meier curves.

### 2.5. Corticosterone Levels in Blood

Circulating corticosterone levels were measured after the restraint stress protocol, but not following the ischemia/reperfusion (I/R) procedure, to ensure that glucocorticoid measurements reflected the effects of stress exposure rather than surgical intervention.

Blood samples were obtained via tail bleeding, as previously described [11]. Mice were restrained on a heating pad and a 1–2 mm piece of the tail tip was removed using sterile scissors. Then the tail was stroked from base to tip to promote blood flow. Approximately 100 µL of blood was collected into a 200 µL EDTA-coated capillary tube (Hopkins Medical Products, Grand Rapids, MI, USA). After collection, gentle pressure was applied with sterile gauze, followed by Blood Stopper Powder (DOGSWELL, St. Louis, MO, USA; Remedy + Recovery) to achieve hemostasis. Once bleeding had ceased, mice were returned to their cages.

Blood samples were centrifuged at 3000 rpm for 15 min at 4 °C, and the plasma was isolated for subsequent analyses. Plasma corticosterone levels (catalog #K014-C1, Arbor Assays, Ann Arbor, MI, USA) were determined using commercially available ELISA kits (Arbor Assays, Ann Arbor, MI, USA), following established protocols. The assay had a sensitivity of 6.71 pg/mL, a 12.8 pg/mL detection limit, and intra- and inter-assay coefficients of variation ranging from 9.4% to 15.1%. All samples were assayed in duplicate according to the manufacturer’s instructions.

### 2.6. Heart Histology

Mice were euthanized and whole hearts were sliced using an acrylic mouse heart slicer matrix. Coronal sections approximately 1.0 mm thick were then allocated to various experimental protocols. For 2,3,5-triphenyltetrazolium chloride (TTC) staining (Catalog T8877, Sigma, St. Louis, MO, USA), the heart slices were incubated in a 1% TTC solution at 37 °C for 10 min, then scanned by placing the slices between two glass slides. In this staining, red indicates viable tissue, while white or pale areas denote infarcted tissue. For consistency and comparability across samples, the mid-ventricular slice at the level of the papillary muscles was used for representative images, as this region provides a reproducible anatomical landmark and typically captures the maximal infarct area. The infarct regions were quantified using ImageJ’s color threshold mode to differentiate between infarcted and viable areas (https://imagej.net/ij/docs/menus/analyze.html (accessed on 21 November 2025)).

For histological analysis, the heart slices were transferred to histological cassettes (Leica Biosystems #3802765, Deer Park, IL, USA) and fixed overnight in 10% formalin. The formalin-fixed tissues were then paraffin-embedded, sectioned at 5 µm thickness, and stained with hematoxylin–eosin (H&E). Masson’s trichrome staining was performed using paraffin-embedded heart sections from the same tissue to assess collagen deposition, with blue staining indicating fibrotic tissue, red staining indicating muscle fibers, and nuclei stained dark purple/black. All histological assessments, including infarct size determination by TTC staining, H&E, and Masson’s trichrome were performed by investigators blinded to genotype and treatment group assignments.

### 2.7. RNA Extraction and Quantitative Real Time-PCR (qRT-PCR)

Total RNA was isolated from tissues and cells using the RNeasy Mini Kit with on-column DNase treatment (Qiagen, Valencia, CA, USA). RNA purity and concentration were assessed using a NanoDrop One spectrophotometer (Thermo Fisher Scientific, Waltham, MA, USA). Target gene expression was measured using the One-Step RT-PCR Universal Master Mix (Thermo Fisher Scientific). qRT-PCR was performed on a CFX96 Real-Time System (Bio-Rad, Hercules, CA, USA) using predesigned TaqMan primer–probe sets for Nfe2l2 (Mm00477784_m1), Gpx2 (Mm00850074_g1), Sod3 (Mm01213380_s1), and the reference gene PPIB (Mm00478295_m1). Thermocycling conditions were 48 °C for 30 min, 95 °C for 10 min, followed by 40 cycles of 95 °C for 15 s and 60 °C for 60 s. Gene expression levels were normalized to PPIB.

### 2.8. Western Blotting

Cardiac tissue was lysed by bead homogenization and sonication in RIPA buffer (Sigma R0278, St. Louis, MO, USA) supplemented with protease (ProBlock™ Gold, GoldBio #GB-108, St. Louis, MO, USA) and phosphatase (Simple Stop™ 1, GoldBio #GB-450, St. Louis, MO, USA) inhibitors. Protein lysates (70–90 μg) were separated on 10% SDS-PAGE gels and transferred to PVDF membranes (Bio-Rad). Membranes were blocked for one hour in Pierce™ Protein-Free Blocking Buffer (Thermo Scientific) and incubated overnight at 4 °C with anti-Nrf2 (ab92946, Abcam; 1:1000, Waltham, MA, USA). Membranes were then incubated with HRP-conjugated secondary antibodies for one hour at room temperature. Signals were detected using Clarity ECL substrate (Bio-Rad) and imaged on a ChemiDoc system.

Membranes were stained for total protein, and lane intensities were quantified in ImageJ. Images were converted to 8-bit, and identical rectangular ROIs were applied to each lane. Using the “Gels” function, lane profiles were generated, and the area under each curve was measured to obtain the total lane signal.

Western blot images were converted to 8-bit, and ROIs were drawn around each Nrf2 band. Integrated density values were measured after background subtraction. Nrf2 signals were normalized to the corresponding total protein values to account for loading differences. Statistical comparisons were confined to genotype-matched littermates of the same sex.

### 2.9. Reactive Oxygen Species and Lipid Peroxidation Measurements

Reactive oxygen species were measured by administering an intraperitoneal injection of dihydroethidium (DHE) (0.3 mg [0.12 mL DMSO and 0.18 mL ddH_2_O]/30 mg mouse), followed by tissue collection 1 h later. Samples were homogenized, extracted with MeOH/HClO_4_, and treated with phosphate buffer (pH 2.6), and the superoxide-specific OH-2E^+^ product was quantified by HPLC. Lipid peroxidation was assessed using a TBARS/MDA colorimetric kit (Arbor Assays, K077-H1, Ann Arbor, MI, USA) according to the manufacturer’s instructions.

### 2.10. Cell Death Assay and Immunohistochemistry for 4-HNE

Cell death was detected using the TUNEL Assay Kit—HRP-DAB (ab206386, Abcam) following the manufacturer’s instructions. This assay allows the recognition of apoptotic nuclei in paraffin-embedded tissue sections. Evaluation of lipid peroxidation by immunohistochemistry for 4-HNE was detected using the following protocol previously described [12]. The slides were de-paraffinized and rehydrated by placing them in Xylene for 5 min twice, 100% ethanol for 5 min twice, 95% ethanol for 4 min twice, and 70% ethanol for 3 min twice. The slides were then rinsed in distilled water for 5 min and samples were encircled using a hydrophobic slide marker. Endogenous peroxidase activity was blocked by incubating the specimen in 100 µL 0.3% H_2_O_2_ in methanol for 20 min at room temperature. The slides were then rinsed in 1X PBS solution twice for 2 min. Then, 100 uL of M.O.M Mouse IgG blocking reagent (Vector Laboratories, MP-2400, Newark, CA, USA) and working solution was applied to slides and incubated for 1 h. The blocking reagent was blotted from the samples and the slides were washed in 1X PBS twice for 2 min. The slides were then incubated in 100 µL M.O.M Normal Horse Serum 2.5% (Vector Laboratories, MP-2400, Newark, CA, USA) for 5 min. Slides were incubated overnight in 100 µL of diluted primary antibody solution (JAI-MNH-020P, Adipogen) at 4 °C. The slides were rinsed in 1X PBS for 2 min twice the next day. The samples were then covered completely with M.O.M ImmPRESS reagent (Vector Laboratories, MP-2400, Newark, CA, USA) for 30 min. The following reagents were added to 5 mL of distilled water: 2 drops of Vector DAB Reagent 1, 4 drops of Vector DAB Reagent 2, and 2 drops of Vector DAB Reagent 3 (Vector Laboratories, SK-4100, Newark, CA, USA). 100 µL of prepared DAB solution was placed on the slides and incubated for 2 min. The slides were then washed in tap water for 5 min and then placed in hematoxylin at room temperature for 1 min. Tap water was used to remove residual hematoxylin. The slides were then dehydrated by placing slides in 95% ethanol for 30 s, 100% ethanol for 30 s twice, and 100% Xylene for 30 s twice. Coverslips were then placed over the tissues with a permanent mounting medium. TUNEL staining, and immunohistochemistry for 4-HNE, were performed by investigators blinded to genotype and treatment group assignments.

Image analysis and quantification for TUNEL were performed using Fiji (ImageJ v1.54f, NIH, Bethesda, MD, USA) [13]. The individual images were separated into individual stain components using the Color Deconvolution plugin with the “H DAB” vector to isolate the DAB channel corresponding to the TUNEL-positive signal. The DAB channel image was converted to 8-bit grayscale, and the contrast was uniformly adjusted across all samples. The DAB channel was inverted so that positively stained regions appeared bright against a dark background. Thresholding was performed using the Otsu algorithm under Image > Adjust > Threshold, with minor manual adjustments applied as needed to include only brown TUNEL-positive nuclei while excluding background and hematoxylin-stained areas. The image was then converted to a binary mask (white = positive signal). The Set Measurements function was configured to record area, mean gray value, and area fraction, with the Limit to threshold option enabled. The total TUNEL-positive area and the total tissue area were measured for each image.

### 2.11. Statistical Analysis

GraphPad Prism v9 was used for all the statistical analyses. Data are presented as mean ± S.E.M. Kaplan–Meier survival curves were generated for NS and S mice after sham or I/R surgery, with 18–22 animals per group. All other experiments used 3–10 mice per group, as detailed in the Materials and Methods section.

Unless otherwise indicated in the figure legends, data were analyzed using an ordinary two-way ANOVA followed by Tukey’s multiple comparisons test to assess differences among treatment groups. The two-way ANOVA evaluated the main effects of sex (male vs. female) and treatment (control vs. stress), as well as their interaction, on the measured parameters. Differences were considered statistically significant at *p* < 0.05.

## 3. Results

### 3.1. Corticosterone Levels Were Elevated in Stressed Female CardioGRKO Mice

**Figure 1:**
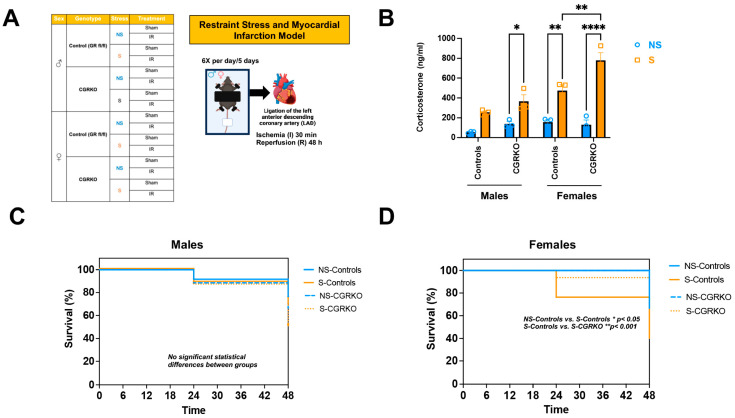
Restraint stress elevates plasma corticosterone levels in mice, and the absence of cardiomyocyte glucocorticoid receptor (GR) improves survival in females following stress and ischemia/reperfusion injury. (**A**) Experimental groups: Male (♂) and female (♀) mice, categorized by genotype: control (GR fl/fl) and CGRKO (lacking cardiomyocyte GR). Mice were further divided into non-stressed (NS, blue) and restraint stress (S, orange) groups. Both NS and S groups were then randomized into sham (no ligation) and ischemia/reperfusion (IR) subgroups. The schematic shows the restraint stress (S) model. Each mouse was placed on a plastic platform with the upper body and limbs secured. The head remained free. After 5 days of stress, mice underwent ischemia followed by 48 h of reperfusion. This figure was created in Biorender. Cruz, D. (2025) https://BioRender.com/tr7wevs (accessed on 14 December 2025). (**B**) Corticosterone measurements: Average plasma corticosterone levels in mice exposed to NS (blue) and S (orange), categorized by sex (male [M] and female [F]), for both control (GR fl/fl) and CGRKO groups. Data represent mean ± S.E. (*n* = 6 independent samples per group). A two-way ANOVA with Tukey’s multiple comparisons test was used to evaluate differences among treatment groups. (**C**,**D**) Kaplan–Meier survival curves: Survival rates of male and female control (solid lines) and CGRKO (dashed lines) mice after ischemia and 48 h reperfusion. Groups include non-stressed (NS, blue) and stressed (S, orange). Sample sizes: NS-control males = 20, NS-control females = 22, S-control males = 20, S-control females = 18, NS-CGRKO males = 19, NS-CGRKO females = 20, S-CGRKO males = 20, S-CGRKO females = 21. * *p* < 0.05, ** *p* < 0.01, **** *p* < 0.0001. Statistical significance was determined using the Mantel–Cox log-rank test with a Bonferroni-corrected threshold. All analyses were conducted using Prism 10 for macOS (Version 10.3.1, build 464).

As shown in Figure 1A, male and female cardioGRKO mice and their littermate controls (with intact GR) were subjected to restraint stress and ischemia/reperfusion (I/R). At baseline (no stress), no statistically significant differences in plasma corticosterone levels were observed between non-stressed-control mice (with intact GR) and non-stressed-cardioGRKO mice, irrespective of sex (Figure 1B). Similarly, corticosterone levels did not differ significantly between non-stressed and stressed male control mice (Figure 1B). In contrast, stressed-male cardioGRKO mice exhibited significantly elevated corticosterone levels compared to their non-stressed counterparts (Figure 1B). Among females, stressed-control mice showed significantly higher corticosterone levels relative to non-stressed-controls (Figure 1B). Furthermore, corticosterone levels in stressed-female cardioGRKO mice were significantly increased compared to their non-stressed counterparts and stressed-female control mice (Figure 1B).

### 3.2. Deletion of Cardiomyocyte GR Leads to Better Survival Rates Following I/R in Female Mice

Survival was monitored in non-stressed and stressed groups of both controls and cardioGRKO mice across both sexes following ischemia/reperfusion (I/R). In male groups, survival rates were similar regardless of genotype or stress exposure (Figure 1C). In contrast, stressed-control females demonstrated a reduced survival rate (~40% at 48 h) compared with non-stressed-controls (~67% survival) and stressed-cardioGRKO females (~82% survival) (Figure 1D).

### 3.3. Deletion of Cardiomyocyte GR Ameliorates I/R Injury in Stressed Female Hearts

**Figure 2:**
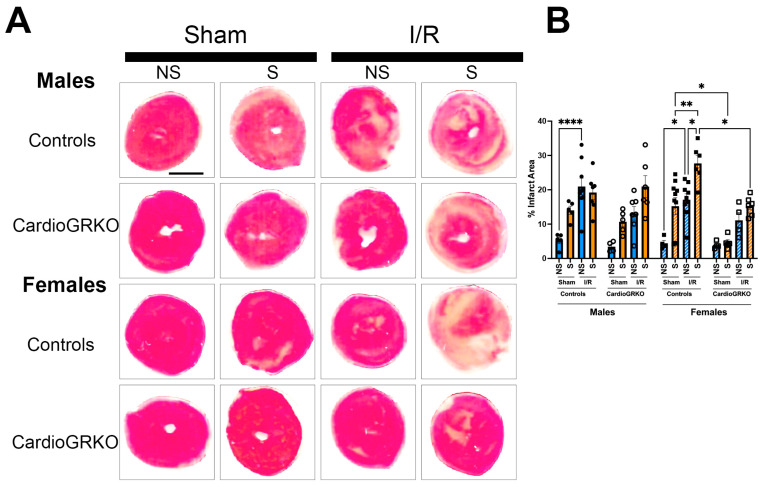
Lack of GR in cardiomyocytes protects the stressed female heart in ischemia/reperfusion. Non-stressed (NS) and stressed (S) male (♂) and female (♀) controls (GR fl/fl) and cardioGRKO (lacking cardiomyocyte GR) mice were subjected to 30 min of ischemia (I) and 48 h of reperfusion (R) following 5 days of restraint stress. (**A**) Representative TTC-stained heart sections from sham and I/R groups. Viable myocardium appears red, and infarcted areas appear beige/white. Scale bar = 12.7 mm. (**B**) Infarct size (%) was quantified in ImageJ using color thresholding. All I/R experiments were performed in females in proestrus (highest estrogen levels). Black circles, male controls and white circles male, cardioGRKO. Black squares, female controls and white squares, female cardioGRKO. A two-way ANOVA with Tukey’s multiple comparisons analysis was used to evaluate differences among group treatments unless otherwise specified. Data represent the mean ± S.E. *n* = 5–10 mice per group. Only statistically significant differences are shown in the figure * *p* < 0.05, ** *p* < 0.01, **** *p* < 0.0001. All analyses were conducted using Prism 10 for macOS (Version 10.3.1, build 464).

In stressed-control females, infarct sizes were significantly larger than those in non-stressed and sham-treated counterparts (Figure 2A,B). In contrast, infarct sizes in stressed-female cardioGRKO hearts did not differ significantly from their non-stressed or sham-treated groups and were notably smaller than those in stressed-control females. In male mice, infarct sizes remained consistent regardless of genotype or stress exposure (Figure 2A,B).

### 3.4. Cardiomyocyte GR Deletion Prevents the Increase in Reactive Oxygen Species Production and Lipid Peroxidation in the Female Heart

**Figure 3:**
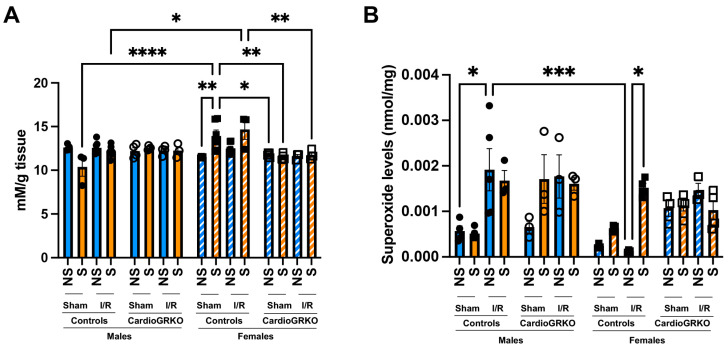
Stress significantly increases heart production of reactive oxygen species in female control hearts with an intact glucocorticoid receptor (GR) signaling, in contrast to female hearts lacking GR in cardiomyocytes. Non-stressed (NS) and stressed (S) male and female controls (GR fl/fl) and cardioGRKO (lacking cardiomyocyte GR) mice (**A**) Lipid peroxidation was measured as malondialdehyde (mM) production per gram (g) of heart tissue. (**B**) Superoxide production levels (nmol per mg of heart tissue). Black circles, male controls and white circles male, cardioGRKO. Black squares, female controls and white squares, female cardioGRKO. A two-way ANOVA with Tukey’s multiple comparisons analysis was used to evaluate differences among group treatments unless otherwise specified. Data represent the mean ± S.E. *n* = 3–7 mice per group. Only statistically significant differences are shown in the figure. * *p* < 0.05, ** *p* < 0.01, *** *p* < 0.001, **** *p* < 0.0001. All analyses were conducted using Prism 10 for macOS (Version 10.3.1, build 464).

Lipid peroxidation levels were significantly elevated in sham-stressed-control females compared to non-stressed females and males (Figure 3A). Although differences between non-stressed and stressed-control females in the context of I/R were not statistically significant, stressed-control females still exhibited higher lipid peroxidation than stressed-males under both sham and I/R conditions. In contrast, no significant increases in lipid peroxidation were observed in the cardioGRKO groups (Figure 3A). Consistently, stress exacerbated superoxide production in the female control hearts during I/R, whereas no changes were noted in the female cardioGRKO hearts (Figure 3B).

### 3.5. Stress Exacerbates Cell Death in the Female Hearts Following I/R and the Absence of Cardiomyocyte GR Protects Both the Male and Female Heart

**Figure 4:**
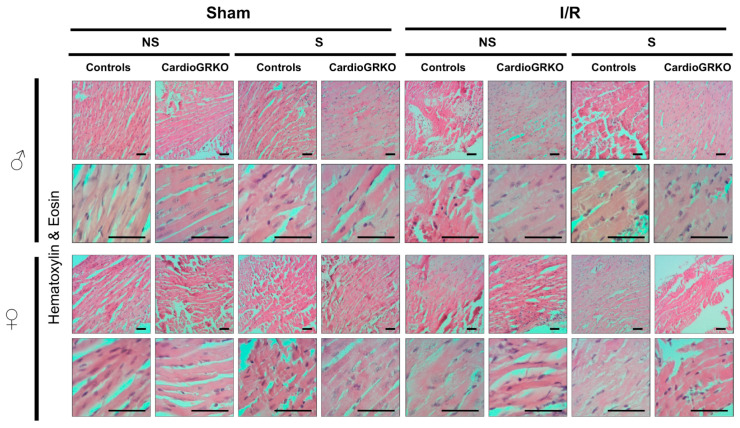
No major morphological differences were observed by Hematoxylin and Eosin staining between male and female hearts across genotypes and treatment. Representative micrographs of H&E-stained heart tissues from non-stressed (NS) and stressed (S) male (♂) and female (♀) controls (GR fl/fl) and cardioGRKO (lacking cardiomyocyte GR) mice that were subjected to sham surgery or 30 min of ischemia (I) and 48 h of reperfusion. Male and female sections were stained at different times; *n* = 5. The scale bar represents 50 µm.

**Figure 5:**
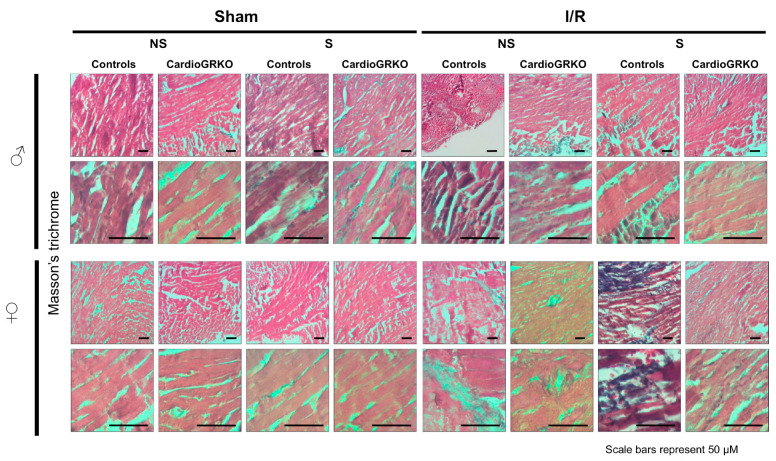
Representative images of Masson’s trichrome staining of heart sections from male (♂) and female (♀) GR fl/fl (control) and cardioGRKO mice under non-stressed (NS) and stressed (S) conditions. Hearts were collected 48 h after 30 min of ischemia (I) or sham surgery. Blue staining indicates collagen deposition and fibrosis. Stressed female control hearts (S GR fl/fl ♀) displayed more prominent collagen accumulation compared to their cardioGRKO counterparts and to male hearts, suggesting greater stress-induced cardiac remodeling in a sex- and genotype-dependent manner. Male and female sections were stained at different times; *n* = 5 per group. Scale bar: 50 µm.

H&E staining revealed signs of inflammation across all I/R experimental groups; however, the groups do not show significant or conclusive histological changes in the sections analyzed (Figure 4). Masson’s trichrome staining showed qualitatively more intense collagen deposition in stressed female control (GR fl/fl) hearts compared with their cardioGRKO counterparts and with male hearts (Figure 5). However, since we did not perform a quantitative assessment, we cannot conclusively state whether one group exhibited greater fibrotic remodeling than another. Moreover, the 48 h post-I/R time point is not optimal for detecting mature fibrotic changes, which typically develop over longer time intervals; therefore, any interpretation of fibrosis at this stage should be considered preliminary.

**Figure 6:**
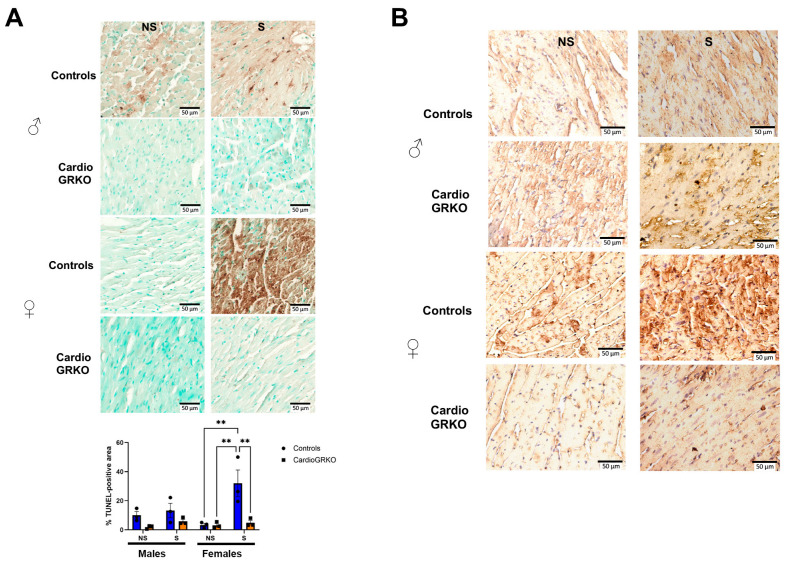
Deletion of cardiomyocyte GR ameliorates cell death in I/R injury in both male and female hearts. (**A**) Representative images of TUNEL staining on the heart sections from no stressed (NS) and stressed (S) male (♂) and female (♀) controls (GR fl/fl) and cardioGRKO (lacking cardiomyocyte GR) mice that were subjected to ischemia (I)/reperfusion (R). Brown color indicates TUNEL positive staining. Cytoplasm TUNEL signal is likely from karyorrhexis (cell fragmentation) during necrotic cell death. TUNEL was performed using the abcam assay kit ab206386, abcam following the manufacturer’s instructions. Quantification of TUNEL-positive area was performed using Fiji (ImageJ) after color deconvolution and thresholding of the DAB channel. Data represent the percentage of TUNEL-positive area relative to total tissue area, averaged from three to five fields per sample. Bars represent mean ± SEM; *n* = (number of animals per group). Statistical significance was determined by one-way ANOVA followed by Tukey’s post hoc test; *p* < 0.05 was considered significant. ** *p* < 0.01. (**B**) Immunohistochemical staining with 4-Hydroxynonenal (4-HNE), a marker of lipid peroxidation and oxidative stress. The 4-HNE staining is presented qualitatively and is supported by the quantitative lipid peroxidation data shown in Figure 3. Representative images of heart sections from NS and S male female controls and cardioGRKO mice that were subjected to I/R. *n* = 3 per group. Scale bar: 50 µm.

TUNEL assay revealed that male and female stressed-control heart sections had significantly more TUNEL-positive staining after I/R compared with their non-stressed counterparts, with stressed control females exhibiting the highest level of cell death (Figure 6A). 4-hydroxynonenal (4-HNE) immunohistochemistry, a marker of lipid peroxidation (15), showed qualitatively more pronounced staining in stressed-control female hearts compared with non-stressed controls or stressed-cardioGRKO females (Figure 6B). Although the images were not quantified and the assessment is qualitative, the increased 4-HNE signal in stressed control females is consistent with the quantitative findings from our lipid peroxidation assays. No differences were observed among the male groups (Figure 6B).

### 3.6. Stress Affects the Cardiac Levels of the Master Regulator of the Antioxidant Response Nrf2 and Downstream Targets in Control Hearts

**Figure 7:**
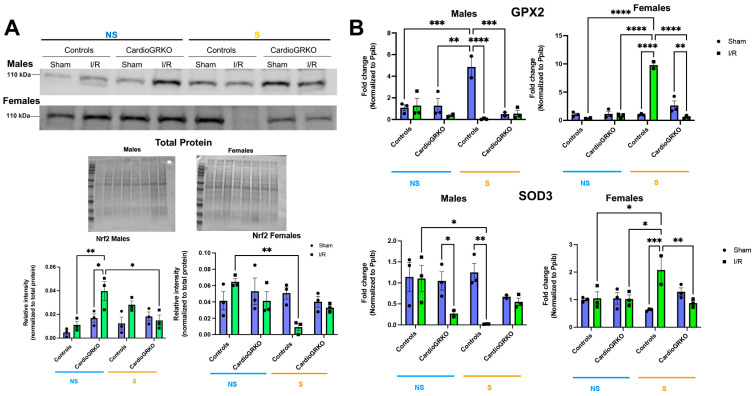
Cardiomyocyte GR maintain the levels and expression of antioxidant mediators in female hearts in response to stress and ischemia/reperfusion. (**A**) Representative Western blot of nuclear factor erythroid 2-related factor 2 (Nrf2) from heart lysates. Protein levels from GR fl/fl and CardioGRKO hearts under non-stressed (N, blue) and stressed (S, orange) conditions with/without (sham) I/R injury. (**B**) Relative mRNA levels of Glutathione Peroxidase 2 (GPX2) and Superoxide Dismutase 3 (SOD3) were measured by qRT-PCR. Data represent the mean ± S.E. *n* = 3 independent samples per group. * *p* < 0.05, ** *p* < 0.01, *** *p* < 0.001, **** *p* < 0.0001. A two-way ANOVA with Tukey’s multiple comparisons analysis was used to evaluate differences among group treatments. All analyses were conducted using Prism 10 for macOS (Version 10.3.1, build 464).

Nuclear factor erythroid 2-related factor 2 (Nrf2), a key regulator of the antioxidant response (16), was significantly reduced in stressed I/R control female hearts relative to non-stressed-controls (Figure 7A). In contrast to males, Nrf2 protein levels remained stable across all conditions in female cardioGRKO hearts. In males, Nrf2 expression increased in non-stressed cardioGRKO hearts following I/R, but significantly decreased in stressed cardioGRKO hearts after I/R. No significant changes in Nrf2 levels were observed in control males under any condition (Figure 7A). We observed some variability in the apparent molecular weight of Nrf2 bands between male and female samples. These differences may be attributed to the use of separate gels for male and female samples, as well as potential sex- and experimental-specific post-translational modifications (e.g., phosphorylation or ubiquitination) that could influence Nrf2 migration on SDS-PAGE.

In females, transcription of the antioxidant genes glutathione peroxidase 2 (GPX2) and superoxide dismutase 3 (SOD3) was significantly upregulated in stressed-control hearts following I/R, compared to non-stressed-controls and stressed-cardioGRKO counterparts (Figure 7B). Conversely, stressed-cardioGRKO females exhibited a significant reduction in GPX2 and SOD3 mRNA levels after I/R. In males, GPX2 transcription increased in stressed-control hearts compared to non-stressed-controls and non-stressed-cardioGRKO groups, but significantly declined in both stressed-I/R cardioGRKO and stressed-I/R control males. SOD3 mRNA levels were markedly reduced in response to I/R in both non-stressed-cardioGRKO and stressed-control male groups, while no changes were detected in non-stressed-control or stressed-cardioGRKO males (Figure 7B).

## 4. Discussion

Mental stress is increasingly recognized as a major contributor to cardiovascular disease (CVD) risk and progression in women [14]. Yet, the mechanisms by which stress exacerbates CVD, particularly in young women, remain poorly understood [15].

In this study, we used cardiomyocyte-specific GR knockout (cardioGRKO) mice in a restraint stress and ischemia/reperfusion (I/R) model to mimic stress and a heart attack and evaluate the contribution of cardiomyocyte GR signaling to cardiac ischemic injury. We found that stressed female cardioGRKO mice were protected from I/R injury, exhibiting improved survival and smaller infarcts relative to stressed female controls. In contrast, stressed male cardioGRKO mice did not differ from their controls, indicating that the detrimental effect of GR signaling during stress is sex-specific.

The lack of GR-dependent effects in stressed males may reflect intrinsic sex differences in hormonal regulation of GR signaling [16]. For example, glucocorticoids acting through GR can directly antagonize estrogen receptor (ER)-mediated gene regulation in both in vitro and in vivo models [17]. Mechanistically, GR can occupy ER-binding sites on target gene promoters, thereby suppressing estrogen-driven gene expression [18]. Therefore, a potential mechanism by which stress may exacerbate ischemic injury in female hearts is via GR antagonism of ER-mediated regulation of cardioprotective genes. Also, GR inhibition of ER-mediated mitochondrial protection may further sensitize female hearts to oxidative damage [19]. Future mechanistic studies using in vitro systems, antioxidant interventions, and in vivo hormonal modulation (e.g., ovariectomy, ER agonists/antagonists) will help to delineate how GR–ER interactions shape oxidative stress responses in the female myocardium.

GR and androgen receptors (AR) share DNA binding elements and cofactors [20], and their actions may be synergistic or antagonistic depending on physiological context [20,21]. Therefore, future studies are also needed to test the role of GR-AR crosstalk in the heart to fully elucidate the molecular and hormonal context of GR signaling in male versus female hearts.

In our study, restraint stress followed by I/R significantly increased superoxide, lipid peroxides, TUNEL staining, and 4-HNE staining in stressed female control hearts, whereas these responses were absent in stressed female cardioGRKO hearts. Because mitochondria are major sources and targets of ROS during I/R, GR-dependent alterations in mitochondrial homeostasis may contribute to this phenotype. Mitochondrial dysfunction is a major contributor to I/R injury through mechanisms involving impaired electron transport, calcium overload, ROS generation, and mitochondrial permeability transition pore (mPTP) opening [22,23,24,25]. GR signaling is known to regulate mitochondrial genes, influence respiratory chain activity, and modulate apoptosis through interactions with Bcl-2 family proteins [26]. While short-term glucocorticoid exposure can enhance mitochondrial function, chronic exposure promotes mitochondrial dysfunction, increases ROS, and heightens cell death susceptibility [27]. However, how GR influences mitochondrial function during cardiac I/R is unknown. Understanding when GR signaling is protective versus detrimental, and identifying key GR-regulated mitochondrial mediators will be critical for understanding stress-induced mitochondrial vulnerability in the female heart.

Our data also show that stressed female control hearts displayed reduced Nrf2 levels after I/R, whereas stressed female cardioGRKO hearts maintained Nrf2 levels comparable to non-stressed controls. Because ROS activate Nrf2 signaling [27,28] and Nrf2 promotes transcription of various antioxidant enzymes, including superoxide dismutase 3 (SOD3) and glutathione peroxidase 2 (GPX2) [27,28]. GPX2 is a selenium-dependent enzyme that reduces lipid peroxides [29], while SOD3 diminishes free radical accumulation [29], with both enzymes playing key roles in cardioprotection [30]. We found that GPX2 mRNA levels were similar between non-stressed groups of both sexes. However, stressed I/R control males showed significantly reduced GPX2 expression compared with sham-stressed males. In contrast, stressed I/R control females showed significantly increased expression of GPX2 and SOD3 compared to non-stressed-controls and stressed-cardioGRKO counterparts. These findings suggest that stress blunts male hearts antioxidant response in I/R injury. In contrast, female hearts exhibit a further enhancement of the antioxidant response under the combined stress and I/R despite reduced Nrf2 levels, potentially as a compensatory mechanism to counterbalance the exacerbated pro-oxidant environment. These sex-specific differences suggest distinct regulatory mechanisms governing antioxidant gene expression in response to stress. Given the critical role of GPX2 and SOD3 in mitigating ROS-mediated myocardial and vascular injury [31,32,33], future studies should investigate the sex-specific role of additional antioxidant pathways in the context of stress and I/R [34,35].

A key limitation of this study is that GPX2 and SOD3 were measured only at the mRNA level. Because transcript abundance does not always reflect protein levels or enzymatic activity [36,37], these results should be interpreted cautiously. Nonetheless, the overall pattern of reduced ROS and preserved Nrf2 in female cardioGRKO hearts supports a model in which, under stress conditions, cardiomyocyte GR suppresses antioxidant defenses in stressed females. Future studies assessing GPX2 and SOD3 protein levels and enzymatic activities will be essential to substantiate this mechanism fully.

We did not measure circulating inflammation makers such as IL-6 or TNFα, which contribute to early reperfusion injury [38], as our focus was on cardiac tissue injury. However, stressed female cardioGRKO mice were protected despite having higher circulating corticosterone, suggesting that the cardiac phenotype in stressed females is not mediated by systemic GR signaling or its potential proinflammatory actions, but rather through cardiomyocyte-specific GR mechanisms.

In the context of inflammation contributing to the phenotype observed in stressed females, it is well established that GR interacts with multiple stress-responsive transcription factors, including AP-1 and NF-κB, which regulate a wide array of inflammatory mediators [39,40,41]. Although GR typically inhibits AP-1 and NF-κB under physiological conditions [42,43], chronic or excessive glucocorticoid exposure can paradoxically enhance inflammatory signaling [44,45,46,47] through mechanisms such as GR desensitization, epigenetic remodeling, altered cofactor balance, and glucocorticoid-induced mitochondrial dysfunction and oxidative stress [48,49]. Estrogen signaling also modulates AP-1 and NF-κB activity [50,51], and ER-mediated inhibition of NF-κB is one established mechanism of female cardioprotection [48,50]. While we did not measure any of these interactions in the present study, we acknowledge that future in vitro studies will be needed to elucidate the molecular mechanisms underlying GR–ER–NF-κB–AP-1 crosstalk in the context of stress and I/R injury.

## 5. Conclusions

Our findings suggest that systemic stress exerts direct, sex-specific effects on the heart, with female hearts showing greater susceptibility to stress-induced injury. This vulnerability appears to be mediated, at least in part, by cardiomyocyte GR, which may impair antioxidant pathways in females.

While our study provides novel insights, it is limited by the small sample size used in some experiments, including Western blots, which may reduce statistical power and limit the generalizability of the findings. We recognize that future studies with larger sample sizes and additional studies measuring protein abundance, enzymatic activity, and transcription factor interactions are needed to fully understand how cardiomyocyte GR shapes antioxidant and inflammatory pathways in a sex-specific manner.

From a translational perspective, our results suggest that GR signaling in the context of stress may directly sensitize female cardiomyocytes to injury; therefore, GR and its downstream signaling may be promising therapeutic targets in women. Selective GR modulators or antagonists already developed for other medical indications could be evaluated in more translational cardiac models, such as human iPSC-derived cardiomyocytes from female donors and organ-on-chip platforms. Additionally, antioxidant-based therapies may offer complementary benefit, as our data suggest that chronic stress suppresses antioxidant defenses, thereby exacerbating cardiac injury in response to ischemia.

Overall, identifying the key mediators that drive stress-induced cardiac injury in females is essential for advancing precision-medicine approaches and guiding the development of targeted GR-based or antioxidant therapies aimed at reducing ischemic heart disease in women experiencing chronic psychosocial stress, which is an important and currently unmet clinical need.

## Data Availability

The authors declare that all supporting data and method descriptions are available within the article or from the corresponding authors upon reasonable request.

## Abbreviations

The following abbreviations are used in this manuscript:

AR	Androgen receptors
CardioGRKO	Cardiomyocyte glucocorticoid receptor knockout
CVD	Cardiovascular disease
DHE	Dihydroethidium
ER α	Estrogen receptor
γ-GCS	γ-glutamylcysteine synthetase
GPX2	Glutathione peroxidase 2
GPX3	Glutathione peroxidase 3
GR	Glucocorticoid receptor
HPA	Hypothalamic–pituitary–adrenal
4-HNE	4-hydroxy-2-nonenal
I/R	Ischemia/Reperfusion
MDA	Malondialdehyde
MI	Myocardial infarction
NS	No stress
Nrf2	Nuclear factor erythroid 2-related factor 2
ROS	Reactive oxygen species
SOD3	Superoxide dismutase 3
S	Stress
